# Exploring the Occurrence Mechanisms of Acute Primary Angle Closure by Comparative Analysis of Ultrasound Biomicroscopic Data of the Attack and Fellow Eyes

**DOI:** 10.1155/2020/8487907

**Published:** 2020-04-24

**Authors:** Fenglei Wang, Dabo Wang, Ling Wang

**Affiliations:** Department of Ophthalmology, The Affiliated Hospital of Qingdao University, Qingdao, China

## Abstract

**Purpose:**

To explore the anatomical characteristics and occurrence mechanisms of acute primary angle closure (APAC) by comparing the quantitative data of UBM images of the APAC and fellow eyes.

**Methods:**

131 patients (262 eyes) were studied over five years by retrospective analysis. The quantitative data from UBM images including angle opening distance at 500 *μ*m (AOD500), trabecular-iris angle (TIA), iris convexity (IC), iris span (IS), iris-lens angle (ILA), iris-lens contact distance (ILCD), iris-ciliary process angle (ICPA), and limbus-ciliary body angle (LCBA) were retrospectively recorded; comparative analysis of the APAC and fellow eyes was performed.

**Results:**

The superior, inferior, nasal, temporal, and mean AOD500, TIA, IC, and LCBA (*P* < 0.001) were significantly smaller in APAC than in fellow eyes. Values of the lens thickness (LT), lens/axial length factor (LAF), lens position (LP), and relative lens position (RLP) were lower in APAC than in fellow eyes (*P* = 0.021; *P* = 0.025; *P* < 0.001; and *P* < 0.001). In APAC eyes, AOD500 was significantly positively correlated with IC, ILCD, and LCBA; TIA was significantly positively significantly correlated with IC, ILCD, and LCBA. In fellow eyes, AOD500 was significantly negatively correlated with ILA and significantly positively correlated with ILCD, ICPA, LCBA, axial length (AL), central anterior chamber depth (CACD), and LP; TIA was significantly negatively correlated with ILA and significantly positively correlated with IS, ILCD, ICPA, LCBA, AL, CACD, LP, and RLP.

**Conclusions:**

Multiple nonpupillary block factors (plateau iris, anterior attachment and insertion of the iris root, anterior shift of the lens, and anterior rotation of the ciliary body) promote the occurrence of APAC, and abnormal positional relationships of the iris, ciliary body, and lens may contribute to APAC.

## 1. Introduction

Glaucoma is the second leading cause of blindness globally, after cataract [1, 2]. It has been estimated that there will be approximately 80 million people with glaucomatous optic neuropathy by 2020 [3, 4]. From population-based epidemiological surveys, the prevalence of angle closure glaucoma (ACG) is much higher in East Asian populations than in European and African populations [5]. Estimates show that primary angle closure glaucoma (PACG) will account for almost 50% of all cases of binocular blindness by 2020 [6].

The Primary Angle Closure Preferred Practice Pattern (PPP) published by the American Academy of Ophthalmology in 2016 [7] states that acute angle closure crisis (AACC) is often accompanied by acute anterior chamber angle blockage and a rapid rise in intraocular pressure (IOP) to extremely high levels, and it may rapidly cause corneal edema (blurred vision, iridizations), moderate pupil dilation, conjunctival hyperaemia, and eyeball pain, which may be accompanied by symptoms such as headache, nausea, and vomiting. The symptoms of AACC may be self-limited, resolved spontaneously, or recurrent. If not promptly treated, AACC may cause permanent vision loss. Fellow eyes of patients with AACC are also at high risk of developing AACC.

Ultrasound biomicroscopy (UBM), a noninvasive high-resolution in vivo anterior imaging technique, has proven to be highly advantageous in assessing the structure of the anterior chamber angle [8]. UBM provides a means for imaging and assessing morphological structures of the anterior segment of the eye (including the ciliary body, the suspensory ligament, and the anterior surface of the lens covered by the iris which cannot be observed during a routine ophthalmic examination). In addition, UBM can be used to perform quantitative and qualitative analyses of pathophysiologic changes in the structures of the anterior segment [9, 10]. In studies on angle closure (AC) diseases, the geometric angle quantification software included in ultrasound biomicroscopes can be utilised to indicate the iris thickness, the ciliary body size, and the anatomical and positional relationships between the iris and the ciliary body.

In the present study, quantitative data acquired from UBM images of the APAC and fellow eyes of APAC patients were consolidated and comparatively analysed. Through the comparison of the anatomical differences in anterior segment structures between the attack and fellow eyes, we investigated the diverse factors involved in the occurrence mechanisms of acute angle closure (AAC).

## 2. Materials and Methods

### 2.1. Materials

#### 2.1.1. Study Population

In total, 131 patients (262 eyes) with monocular APAC who sought medical consultation and were hospitalised for treatment at the Department of Ophthalmology, The Affiliated Hospital of Qingdao University in Qingdao, China, between October 2013 and October 2018 were included in the study. The subjects consisted of 110 women (220 eyes, 83.97%) and 21 men (42 eyes, 16.03%) with a mean age of 66.09 ± 8.52 years.

Two groups were established, with the APAC eyes of the 131 patients (131 eyes) belonging to the case group and the fellow eyes (131 eyes) belonging to the control group. All patients had signed informed consent forms for hospitalisation, surgery, and clinical trial-related matters upon hospital admission (if patients were unable to provide their signatures due to a lack of legal capacity, illiteracy, or visual impairment, the informed consent forms were signed by a direct relative). All procedures of this study were conducted in compliance with the Declaration of Helsinki and were reviewed and approved for reference by the Ethics Committee of The Affiliated Hospital of Qingdao University. The clinical study was purely academic with no involvement of commercial activities.

#### 2.1.2. Inclusion and Exclusion Criteria [11]

(1) No major underlying disease requiring medical or surgical intervention (excluding patients with hypertension, diabetes, dialysis for renal failure, immune diseases requiring long-term oral hormone treatment, and long-term chemotherapy after surgery for a malignant tumor)
(2) Uniocular acute angle closure glaucoma, with the time of onset less than 5 days. Patients with previous history of acute angle closure attack (e.g., old pigmental keratic precipitate, segmental atrophy of the iris, and old glaucomatous fleck of the lens observed) were excluded. Before admission, neither of the eyes has received medication, laser, or surgical intervention (including anterior chamber puncture treatment)
(3) Ophthalmic examination does not reveal any ophthalmic diseases affecting the chamber angle such as iris root detachment, anterior chamber angle recession, space-occupying lesions in the anterior and posterior ocular segments, suprachoroidal effusion (ciliary body or choroidal detachment), retinal detachment, and acute or old uveitis
(4) Presence of typical characteristics of AACC [12] during disease onset, such as
  - presence of at least one of the following symptoms: periocular pain, headache, nausea, vomiting, decreased visual acuity, and/or a history of intermittent iridization attacks
  - IOP ≥ 21 mmHg (measured by a Goldmann applanation tonometer)
  - the contact range of angle trabecular observed under a gonioscope exceeded 180
  - presence of at least four abnormal eye signs observed under a slit lamp: ciliary congestion, corneal endothelial edema, fixed medium-sized pupil, glaucomatous fleck, and shallow peripheral anterior chamber
(5) Excluding patients with allergy to the mydriatic agent (compound tropicamide eye drops: eye drops containing 0.5% tropicamide and 0.5% phenylephrine hydrochloride) and surface anesthetic agent (oxybuprocaine hydrochloride eye drops: 0.4% oxybuprocaine solution, 20 mL : 80 mg)
(6) Excluding patients with poor image clarity in UBM, A-Scan, and other imaging examinations which cannot clearly distinguish the anatomical structures and morphological characteristics
(7) Excluding patients with incomplete clinical data, as this makes later data statistics and analysis extremely difficult

### 2.2. Methods

#### 2.2.1. General Ophthalmic Examination

Patients were inquired about their medical history, and it was recorded in detail for all subjects after admission.

Ophthalmic examination included computer optometry (Topcon Ltd., Model KR-8900, Japan), best corrected visual acuity (Topcon Ltd., Model CV-5000, Japan), intraocular pressure (Goldmann applanation tonometer), slit lamp, and related examinations (preset lens, gonioscopy) (Haag-Streit Ltd., Model BM 900, Switzerland).

A-Scan (Quantel Medical Ltd., Model Aviso, France) was used to measure axial length (AL), central anterior chamber depth (CACD), and lens thickness (LT). The parameters of lens position should be used in the study, which can be obtained indirectly through the above data calculation: lens/axial length factor (LAF) = LT/AL∗10; lens position (LP) = CACD + 1/2LT; and relative lens position (RLP) = LP/AL∗10. [13–16]

Ultrasound biomicroscopy (Suoer Electronic Ltd., Model SW3200L, China) was used to measure relevant parameters [13, 17] (see below for details).

#### 2.2.2. UBM Imaging Quantitative Data Acquisition Method

1. *Angle Opening Distance (Angle Opening Distance at 500 μm from the Scleral Spur, AOD500)* [16]. The specific measurement method was to start at a point 500 *μ*m from the scleral spur along the corneal endothelium surface and make a line perpendicular to the corneal endothelium through this point. The perpendicular line intersected with the anterior iris surface. This vertical line was AOD500. This parameter can indirectly reflect the degree of the chamber angle opening
2. *Trabecular-Iris Angle (TIA)* [18]. The clinical TIA value was consistent with the anterior chamber angle of 500 *μ*m (anterior chamber angle at 500 *μ*m from the scleral spur, ACA500). The specific measurement method was to make a triangle with AOD500 as the base and the recess at the iris root as the vertex, and the included angle of the vertex was TIA. This parameter can indirectly reflect the degree of the chamber angle opening
3. *Iris Convexity (IC)* [19–21]. Iris convexity is the curvature of the posterior surface of the iris and is indirectly expressed by the length of the vertical line from the most protruding position of the iris to the line connecting the iris root and the iris apex [18, 22–24]. A positive value of IC represented forward convexity of the iris, and a negative value represented posterior iris bombe. For the iris with both anterior and posterior bombe, the direction with greater bombe was taken
4. *Iris Span (IS)*. The straight line distance from the attachment point of the root of the posterior iris surface to the iris apex (the iris apex is the midpoint of the iris-lens contact distance (ILCD)). This parameter can directly reflect the average distance that the iris extends to the central part of the eyeball and indirectly reflects the size of the pupil
5. *Iris-Lens Angle (ILA)* [18]. The specific measurement method was to take the contact point between the posterior iris surface and the anterior lens surface as the vertex, and two sides along this vertex were tangent lines of the posterior iris surface and the anterior lens surface, respectively. The included angle formed was ILA. This parameter can directly reflect the relative position of the lens and central iris and indirectly reflect the degree of attachment and detachment of the lens and iris
6. *Iris-Lens Contact Distance (ILCD)* [18]. The line between the contact points of the anterior and posterior iris surfaces and the anterior lens surface. This parameter can directly reflect the degree of attachment and detachment of the lens and iris and indirectly reflect the relative positions of the two
7. *Iris-Ciliary Process Angle (ICPA)*. The angle between the root of the posterior surface of the iris and the anterior surface of the ciliary process. This parameter can directly reflect the positional relationship between the ciliary process and the iris root
8. *Limbus-Ciliary Body Angle (LCBA)*. The two sides of the angle are, respectively, the extension line of the connection line from the central point of the ciliary process to the central point of the ciliary body basement and the extension of the connection line between the central point of limbal thickness and the central point of one-third thickness of the lateral part of the cornea along the direction of the long axis of the ciliary body. The two sides can reflect the average trend of the ciliary body and corneal limbus, and this included angle can directly reflect the positional relationship and degree of separation (pronation or supination) between the ciliary body and the corneal limbus. It can also reflect the relative position of the whole ciliary body inside the eyeball

**Figure 1:**
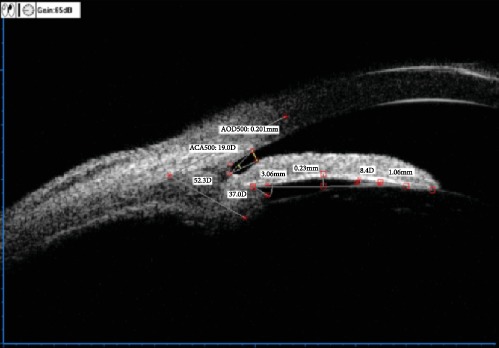
Standardised collection of quantitative data from ocular UBM images.

Figure 1 is a local image of the nasal quadrant of the left eye of a UBM scanning case. The manual labeling and calculation of quantitative data were completed by using UBM's own labeling software and then directly obtaining output (the specific output data were as follows: AOD500 = 0.201 mm; ACA500 (TIA) = 19.0D; IC = 0.23 mm; IS = 3.06 mm; ILA = 8.4D; ILCD = 1.06 mm; LCPA = 37.0D; and LCBA = 52.3D).

### 2.3. Statistical Analysis

Data analysis was performed using SPSS 20.0. The paired *t*-test was used for comparative analysis of the quantitative data obtained from UBM images and A-Scan images of APAC eyes and fellow eyes. Univariate linear regression analysis was performed to determine the respective relationships of AOD500 and TIA of the attack and fellow eyes with other quantitative parameters; the regression formulae and *R* values were determined to generate univariate scatter plots. Differences were considered statistically significant when *P* < 0.05.

## 3. Results

### 3.1. Comparative Analysis of Quantitative Data from UBM Images of the Case and Control Groups

Table 1 indicates that the superior, inferior, nasal, temporal, and global AOD500 of the case group were significantly smaller than those of the control group (*P* < 0.001, 95% CI: -0.07–-0.05, -0.09–-0.06, -0.11–-0.08, -0.12–-0.09, and -0.09–-0.08).

The superior, inferior, nasal, temporal, and global TIA of the case group were significantly smaller than those of the control group (*P* < 0.001, 95% CI: -6.13–-3.83, -7.74–-5.21, -9.90–-7.04, -11.18–-8.13, and -8.08–-6.71).

The superior, inferior, nasal, temporal, and global IC of the case group were significantly smaller than those of the control group (*P* < 0.001, 95% CI: -0.12–-0.07, -0.15–-0.10, -0.13–-0.08, -0.13–-0.07, and -0.12–-0.09).

The superior, inferior, nasal, temporal, and global IS of the case group were significantly shorter than those of the control group (*P* = 0.002, 95% CI: -0.31–-0.07; *P* < 0.001, 95% CI: -0.15–-0.10, -0.39–-0.19, -0.46–-0.27, and -0.35–-0.24).

The superior, nasal, and global ILA of the case group were significantly smaller than those of the control group (*P* = 0.001, 95% CI: -3.54–-0.93; *P* = 0.001, 95% CI: -3.41–-0.87; and *P* = 0.015, 95% CI: -2.00–0.65), but no significant differences were observed in the inferior and temporal ILA (*P* = 0.208, 95% CI: -2.29–0.50; *P* = 0.978, 95% CI: -1.45–1.41).

The superior, inferior, and global ILCD of the case group were significantly longer than those of the control group (*P* = 0.039, 95% CI: 0.00–0.18; *P* = 0.002, 95% CI: 0.06–0.27; and *P* < 0.001, 95% CI: 0.05–0.15), but no significant differences were observed in the nasal and temporal ILCD (*P* = 0.327, 95% CI: -0.06–0.18; *P* = 0.138, 95% CI: -0.02–0.18).

The global ICPA of the case group was significantly smaller than that of the control group (*P* < 0.001, 95% CI: -5.96–-0.63), but no significant differences were observed in the superior, inferior, nasal, and temporal ICPA (*P* = 0.455, 95% CI: -6.68–3.01; *P* = 0.229, 95% CI: -7.90–1.90; *P* = 0.075, 95% CI: -10.86–0.52; and *P* = 0.291, 95% CI: -9.14–2.76).

The superior, inferior, nasal, temporal, and global LCBA of the case group were significantly smaller than those of the control group (*P* < 0.001, 95% CI: -6.14–-2.11; *P* = 0.006, 95% CI: -5.18–-0.88; *P* = 0.013, 95% CI: -6.38–-0.77; *P* = 0.006, 95% CI: -5.50–-0.95; and *P* < 0.001, 95% CI: -4.64–-2.33).

### 3.2. Comparative Analysis of Ocular Biological Parameters of the Case and Control Groups Measured from A-Scans

Table 2 indicates that the values of LT, LAF, LP, and RLP of the case group were significantly lower than those of the control group (*P* = 0.021, 95% CI: -0.21–-0.02; *P* = 0.025, 95% CI: -0.10–-0.01; *P* < 0.001, 95% CI: -0.16–-0.06; and *P* < 0.001, 95% CI: -0.07–-0.03). However, there were no significant differences in AL and CACD between the two groups (*P* = 0.943, 95% CI: -0.09–0.09; *P* = 0.051, 95% CI: -0.10–0.00).

### 3.3. Univariate Linear Regression Analysis of AOD500/TIA and Other Anterior Segment Parameters

Table 3 indicates the results of univariate linear regression analysis of AOD500 and other anterior segment parameters of the case and control groups, which are summarised as follows.

In the case group, AOD500 was significantly positively correlated with IC, ILCD, and LCBA (*P* = 0.001, *R* = 0.137; *P* < 0.001, *R* = 0.244; and *P* < 0.001, *R* = 0.144) and not significantly correlated with IS, ILA, ICPA, AL, CACD, LT, LAF, LP, and RLP (*P* = 0.084, *R* = 0.070; *P* = 0.435, *R* = 0.031; *P* = 0.081, *R* = 0.069; *P* = 0.593, *R* = 0.043; *P* = 0.978, *R* = 0.002; *P* = 0.774, *R* = 0.023; *P* = 0.719, *R* = 0.029; *P* = 0.793, *R* = 0.021; and *P* = 0.647, *R* = 0.036).

In the control group, AOD500 was significantly negatively correlated with ILA (*P* < 0.001, *R* = 0.178); significantly positively correlated with ILCD, ICPA, LCBA, AL, CACD, and LP (*P* < 0.001, *R* = 0.350; *P* = 0.004, *R* = 0.113; *P* = 0.045, *R* = 0.158; *P* = 0.033, *R* = 0.168; *P* = 0.008, *R* = 0.208; and *P* < 0.001, *R* = 0.284); and not significantly correlated with IC, IS, LT, LAF, and RLP (*P* = 0.160, *R* = 0.056; *P* = 0.060, *R* = 0.075; *P* = 0.273, *R* = 0.087; *P* = 0.870, *R* = 0.013; and *P* = 0.066, *R* = 0.146).

Table 4 indicates the results of univariate linear regression analysis of TIA and other anterior segment parameters of the case and control groups, which are summarised as follows.

In the case group, TIA was significantly positively correlated with IC, ILCD, and LCBA (*P* < 0.001, *R* = 0.142; *P* < 0.001, *R* = 0.247; and *P* < 0.001, *R* = 0.139) and not significantly correlated with IS, ILA, ICPA, AL, CACD, LT, LAF, LP, and RLP (*P* = 0.090, *R* = 0.068; *P* = 0.380, *R* = 0.035; *P* = 0.105, *R* = 0.064; *P* = 0.547, *R* = 0.048; *P* = 0.885, *R* = 0.012; *P* = 0.916, *R* = 0.008; *P* = 0.839, *R* = 0.016; *P* = 0.977, *R* = 0.002; and *P* = 0.830, *R* = 0.017).

In the control group, TIA was significantly negatively correlated with ILA (*P* < 0.001, *R* = 0.175); significantly positively correlated with IS, ILCD, ICPA, LCBA, AL, CACD, LP, and RLP (*P* = 0.050, *R* = 0.078; *P* < 0.001, *R* = 0.346; *P* = 0.003, *R* = 0.119; *P* < 0.001, *R* = 0.173; *P* = 0.049, *R* = 0.156; *P* = 0.008, *R* = 0.209; *P* < 0.001, *R* = 0.285; and *P* = 0.048, *R* = 0.157); and not significantly correlated with IC, LT, and LAF (*P* = 0.197, *R* = 0.051; *P* = 0.274, *R* = 0.087; and *P* = 0.821, *R* = 0.018).

### 3.4. Comparative Analysis and Scatter Plots of Linear Regression Results

**Figure 2:**
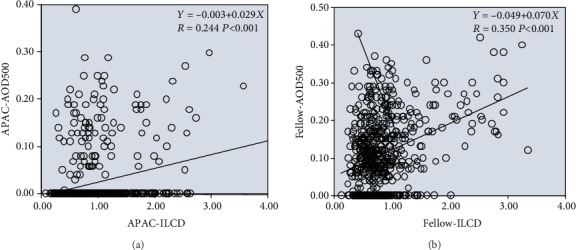
Linear regression relationships between AOD500 and ILCD in the APAC (a) and fellow (b) eyes.

Univariate linear regression analysis indicated that AOD500 and ILCD were significantly positively correlated in both the case and control groups (for the case group, regression formula: *Y* = 0.003 + 0.029*X*, *R* = 0.244, *P* < 0.001; for the control group, regression formula: *Y* = 0.049 + 0.070*X*, *R* = 0.350, *P* < 0.001); i.e., as ILCD increased, AOD500 increased and the chamber angle widened. However, the positive correlation between AOD500 and ILCD was stronger in the control group than in the case group (Figure 2).

**Figure 3:**
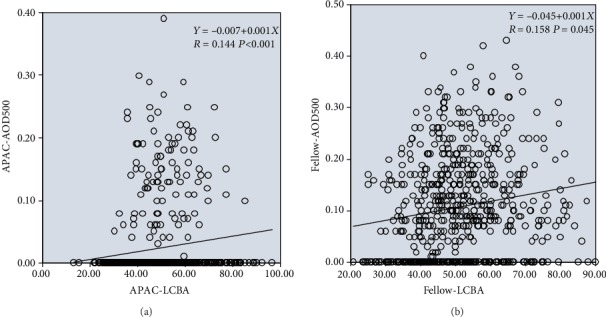
Linear regression relationships between AOD500 and LCBA in the APAC (a) and fellow (b) eyes.

AOD500 and LCBA were significantly positively correlated in both the case and control groups (for the case group, regression formula: *Y* = 0.007 + 0.001*X*, *R* = 0.144, *P* < 0.001; for the control group, regression formula: *Y* = 0.045 + 0.001*X*, *R* = 0.158, *P* = 0.045); i.e., as LCBA increased, AOD500 increased and the chamber angle widened. The strengths of the positive correlation between AOD500 and LCBA were similar in the case and control groups (Figure 3).

**Figure 4:**
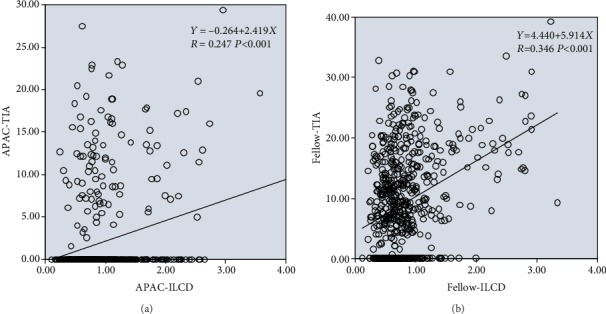
Linear regression relationships between TIA and ILCD in the APAC (a) and fellow (b) eyes.

TIA and ILCD were significantly positively correlated in both the case and control groups (for the case group, regression formula: *Y* = 0.264 + 2.419*X*, *R* = 0.247, *P* < 0.001; for the control group, regression formula: *Y* = 4.440 + 5.914*X*, *R* = 0.346, *P* < 0.001); i.e., as ILCD increased, TIA increased and the chamber angle widened. However, the positive correlation between TIA and ILCD was stronger in the control group than in the case group (Figure 4).

**Figure 5:**
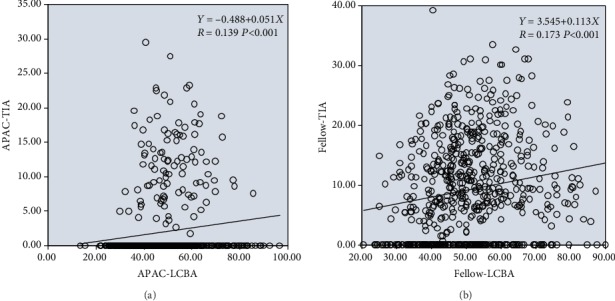
Linear regression relationships between TIA and LCBA in the APAC (a) and fellow (b) eyes.

TIA and LCBA were significantly positively correlated in both the case and control groups (for the case group, regression formula: *Y* = 0.488 + 0.051*X*, *R* = 0.139, *P* < 0.001; for the control group, regression formula: *Y* = 3.545 + 0.113*X*, *R* = 0.173, *P* < 0.001); i.e., as LCBA increased, TIA increased and the chamber angle widened. The strengths of the positive correlation between TIA and LCBA were similar in the case and control groups (Figure 5).

**Figure 6:**
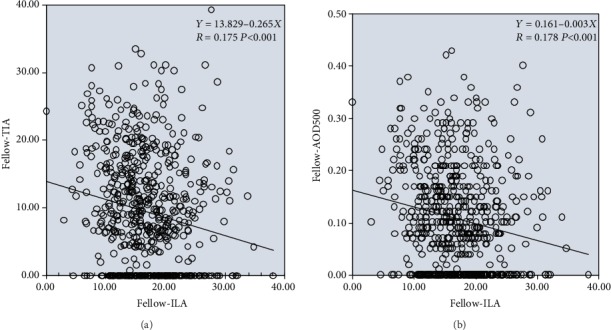
Linear regression relationships between ILA and TIA (a)/AOD500 (b) in fellow eyes.

In the control group, both TIA and AOD500 were significantly negatively correlated with ILA (for the relationship between TIA and ILA, regression formula: *Y* = 13.829 − 0.265*X*, *R* = 0.175, *P* < 0.001; for the relationship between AOD500 and ILA, regression formula: *Y* = 0.161 − 0.003*X*, *R* = 0.178, *P* < 0.001); i.e., in the control group, as ILA increased, both TIA and AOD500 decreased and the chamber angle narrowed. The strengths of the negative correlations of TIA/AOD500 and ILA were similar in the control group (Figure 6).

## 4. Discussion

In the existing literature, a multitude of studies on the occurrence mechanisms of APAC have been reported; however, the results obtained by researchers have varied. Shabana et al. and Kwon et al. [25, 26] utilised anterior segment optical coherence tomography (AS-OCT) to analyse the images of patients with PAC and classified angle closure mechanisms into four categories: (1) pupillary block (PB): bowing of the iris into a convex form, accompanied by a shallow central anterior chamber depth; (2) plateau iris configuration (PIC): the peripheral iris rises from the root and is extremely close to the trabecular wall of the chamber angle; at a certain point, there is a sharp turn of the iris away from the chamber angle, accompanied by a flat central iris and a relatively deep central anterior chamber; (3) thick peripheral iris roll (TPIR): a relatively thick iris with significant peripheral circumferential folds and a chamber angle occupying a higher proportion of space; and (4) exaggerated lens vault (ELV): the lens pushes the iris anteriorly, resulting in a shallower anterior chamber and narrower chamber angle, which is clinically known as the “volcano-like configuration.” Their results indicated that the most common occurrence mechanism of APAC was PB (35%), followed by TPIR (26%), PIC (23%), and ELV (17%). Through an analysis of UBM images, Suwan et al. [27] found that besides the PB mechanism (23.6%), the main underlying nonpupillary block mechanism was the antedisplacement of the lens-iris diaphragm (including the crowded-angle mechanism and anterior lens subluxation mechanism) (68.1%), followed by the plateau iris mechanism. In another study, Wang et al. [28] utilised UBM for the observation of anatomical structures related to the chamber angle and they found that the angle closure mechanisms for PACG can be classified as follows: (1) pupillary block factors (38.1%), (2) nonpupillary block factors (7.1%), and (3) combination of multiple mechanisms (54.8%). The majority of PAC cases in China occur due to a combination of multiple mechanisms. Besides pupillary block factors, nonpupillary block factors such as the anterior insertion of the iris root, forward rotation of the ciliary body, and thick peripheral iris exist in most patients, resulting in an unusual form of angle closure known as creeping angle closure. Therefore, there is a need to focus research efforts on the theory that angle closure is promoted by the joint action of multiple angle closure mechanisms.

The results of our study (Table 1) provide further evidence in support of the aforementioned viewpoints. The iris convexity (IC) of the case group (APAC eyes) was significantly smaller than that of the control group (fellow eyes), which shows that pupillary block factors did not constitute the main mechanism of AAC. The AOD500 and TIA of the case group were significantly smaller than those of the control group, indicating a more anterior insertion of the iris root, narrower anterior chamber angle, and higher proportion of the plateau iris configuration (PIC) in the case group. The iris span (IS) of the case group was significantly shorter than that of the control group, indicating that the iris was relatively shorter and smaller in the case group, leading to the development of more circumferential folds and a higher tendency of peripheral iris bunching, which resulted in the blocking of the chamber angle. The limbus-ciliary body angle (LCBA) of the case group was significantly smaller than that of the control group. This is also indicative of the more anterior position of the ciliary body within the eye, which resulted in a greater anterior push on the peripheral iris and a higher tendency of acute closure of the anterior chamber angle.

Although the lens thickness (LT) of the case group was significantly smaller than that of the control group (Table 2), the values of parameters that reflect the relative lens position, i.e., lens/axial length factor (LAF), lens position (LP), and relative lens position (RLP), were also lower in the case group. This shows that, compared with the control group, the lens of the case group was in a more anterior position within the eye. Consequently, the ratio of the lens and its anterior space to the entire axial length was smaller, which led to a higher tendency of chamber angle crowding. In addition, the iris-lens angle (ILA) of the case group was significantly smaller than that of the control group (Table 1), but the iris-lens contact distance (ILCD) was significantly longer in the case group than in the control group. Based on the changes in these two quantitative parameters, it can be further deduced that antedisplacement of the lens-iris diaphragm occurred in the case group, giving rise to a volcano-like configuration and aggravating the progression of acute angle closure.

Our study has proven again that pupillary block factors do not constitute the main occurrence mechanism of APAC, whereas the joint participation of multiple nonpupillary block factors promotes the occurrence and progression of APAC. During our clinical observations, it was also found that the administration of a miotic agent in patients with such an occurrence mechanism aggravated their condition, which further supports the above viewpoint. Furthermore, we also observed that the iris convexity of fellow eyes was larger, which indicates that pupillary block factors formed the dominant mechanism of the narrowing or blocking of the chamber angle. Therefore, it may be possible that the pupillary block is a common anatomical characteristic of both the attack and fellow eyes, but the joint action of the pupillary block and multiple nonpupillary block factors in the attack eye mediates and promotes the occurrence of AAC.

For the case group and the control group with a normal lens position, an increase in the iris-lens contact distance (ILCD) indicates that the length of the iris covering the anterior surface of the lens increased, i.e., the iris span increased. This would lead to the widening of the anterior chamber angle if all other quantitative parameters remained constant, resulting in a miosis-like effect. Therefore, ILCD was positively correlated with AOD500 and TIA in both the case and control groups. Under conditions of a relatively posterior lens position and an equivalent centripetal extension of the iris (ILCD is increased by the same extent) in the control group, the range and degree of anterior chamber angle widening would be increased. Consequently, the correlations between ILCD and AOD500/TIA were stronger in the control group than in the case group (Figures 2 and 4).

In both the case and control groups, an increase in the limbus-ciliary body angle (LCBA) indicated that the ciliary body occupied a more posterior position in the eye, consequently reducing the force of the anterior push on the iris, increasing the degree of anterior chamber angle widening, and lowering the possibility of AAC. This is consistent with the positive correlations shown in Figures 3 and 5.

In the control group, pupillary block factors were dominant and the proportion of convex irises was higher. Therefore, the magnitude of the iris-lens angle (ILA) reflected the degree of convexity of the iris to a certain extent. As ILA increased, iris convexity increased and the anterior chamber angle narrowed. This provides a reasonable explanation for the negative correlations between ILA and AOD500/TIA in the control group shown in Figure 6.

Compared to the studies on APAC mechanism through UBM imaging before, our study had the following advantages. Firstly, we identified the study subjects as APAC eyes and fellow eyes, which could more directly reflect the relationship between structural differences and acute attacks. Secondly, our study was a comparative analysis of quantitative data. By combining various parameters of the lens displayed by A ultrasound with those of the iris and ciliary body revealed by UBM, we could better explain the morphological and anatomical characteristics of APAC. Finally, this study belonged to the second part of the overall study. The first part was the comparative analysis of the qualitative parameters of UBM, and the third part was the discussion of the mechanism of acute angle closure secondary to lens subluxation [11].

Although it is an undeniable fact that choroidal factors also play a key role in the occurrence mechanisms of APAC [29], we were unfortunately unable to include choroidal data in this retrospective study due to the lack of standardised measurement protocols. In our future studies, standardised measurement and observation indicators will be established to substantiate our research on the occurrence mechanisms of APAC.

In summary, we found that multiple nonpupillary block factors (plateau iris, anterior attachment and insertion of the iris root, anterior shift of the lens, and anterior rotation of the ciliary body) promote the occurrence of APAC, and abnormal positional relationships of the iris, ciliary body, and lens may contribute to APAC.

## Figures and Tables

**Table 1:** Comparisons on the quantitative data of UBM images in the APAC and fellow eyes $\left(\bar{x}\pm s\right)\left(n\right)$.

	Data from UBM images			
	APAC	Fellow	*P*	95% CI
AOD500 ± *s*(*n*)				
Superior	0.01 ± 0.03 (131)	0.07 ± 0.09 (131)	0.001^∗^	-0.07~-0.05
Inferior	0.01 ± 0.04 (131)	0.09 ± 0.09 (131)	0.001^∗^	-0.09~-0.06
Nasal	0.02 ± 0.04 (131)	0.12 ± 0.10 (131)	0.001^∗^	-0.11~-0.08
Temporal	0.05 ± 0.08 (131)	0.15 ± 0.10 (131)	0.001^∗^	-0.12~-0.09
Global quadrant	0.02 ± 0.06 (524)	0.11 ± 0.10 (524)	0.001^∗^	-0.09~-0.08
TIA ± *s*(*n*)				
Superior	0.69 ± 2.58 (131)	5.68 ± 7.05 (131)	0.001^∗^	-6.13~-3.83
Inferior	1.21 ± 3.74 (131)	7.69 ± 7.21 (131)	0.001^∗^	-7.74~-5.21
Nasal	1.97 ± 4.91 (131)	10.44 ± 8.39 (131)	0.001^∗^	-9.90~-7.04
Temporal	3.89 ± 6.88 (131)	13.54 ± 8.46 (131)	0.001^∗^	-11.18~-8.13
Global quadrant	1.94 ± 4.94 (524)	9.34 ± 8.33 (524)	0.001^∗^	-8.08~-6.71
IC ± *s*(*n*)				
Superior	0.22 ± 0.14 (129)	0.31 ± 0.11 (129)	0.001^∗^	-0.12~-0.07
Inferior	0.24 ± 0.15 (129)	0.36 ± 0.13 (129)	0.001^∗^	-0.15~-0.10
Nasal	0.16 ± 0.11 (131)	0.27 ± 0.10 (131)	0.001^∗^	-0.13~-0.08
Temporal	0.24 ± 0.13 (131)	0.34 ± 0.13 (131)	0.001^∗^	-0.13~-0.07
Global quadrant	0.21 ± 0.14 (520)	0.32 ± 0.12 (520)	0.001^∗^	-0.12~-0.09
IS ± *s*(*n*)				
Superior	3.47 ± 0.68 (129)	3.67 ± 0.53 (129)	0.002	-0.31~-0.07
Inferior	3.57 ± 0.78 (129)	3.90 ± 0.54 (129)	0.001^∗^	-0.45~-0.21
Nasal	3.07 ± 0.58 (131)	3.36 ± 0.50 (131)	0.001^∗^	-0.39~-0.19
Temporal	3.31 ± 0.63 (131)	3.66 ± 0.51 (131)	0.001^∗^	-0.46~-0.27
Global quadrant	3.36 ± 0.70 (520)	3.65 ± 0.55 (520)	0.001^∗^	-0.35~-0.24
ILA ± *s*(*n*)				
Superior	15.58 ± 7.72 (131)	17.81 ± 5.22 (131)	0.001	-3.54~-0.93
Inferior	16.22 ± 7.77 (131)	17.11 ± 5.37 (131)	0.208	-2.29~0.50
Nasal	13.41 ± 6.70 (131)	15.55 ± 5.74 (131)	0.001	-3.41~-0.87
Temporal	17.35 ± 7.45 (131)	17.37 ± 5.63 (131)	0.978	-1.45~1.41
Global quadrant	15.64 ± 7.54 (524)	16.96 ± 5.50 (524)	0.015	-2.00~0.65
ILCD ± *s*(*n*)				
Superior	0.87 ± 0.40 (129)	0.77 ± 0.42 (129)	0.039	0.00~0.18
Inferior	0.94 ± 0.57 (129)	0.77 ± 0.49 (129)	0.002	0.06~0.27
Nasal	0.98 ± 0.53 (131)	0.92 ± 0.57 (131)	0.327	-0.06~0.18
Temporal	0.91 ± 0.51 (131)	0.84 ± 0.47 (131)	0.138	-0.02~0.18
Global quadrant	0.92 ± 0.51 (520)	0.83 ± 0.49 (520)	0.001^∗^	0.05~0.15
ICPA ± *s*(*n*)				
Superior	37.07 ± 26.16 (131)	38.90 ± 26.50 (131)	0.455	-6.68~3.01
Inferior	43.24 ± 22.47 (131)	46.23 ± 25.52 (131)	0.229	-7.90~1.90
Nasal	44.44 ± 30.64 (131)	49.61 ± 28.70 (131)	0.075	-10.86~0.52
Temporal	45.26 ± 29.55 (131)	48.45 ± 27.30 (131)	0.291	-9.14~2.76
Global quadrant	42.50 ± 27.52 (524)	45.80 ± 27.29 (524)	0.001^∗^	-5.96~-0.63
LCBA ± *s*(*n*)				
Superior	40.79 ± 11.47 (131)	44.92 ± 11.90 (131)	0.001^∗^	-6.14~-2.11
Inferior	47.00 ± 11.18 (131)	50.03 ± 10.88 (131)	0.006	-5.18~-0.88
Nasal	51.62 ± 14.54 (131)	55.20 ± 13.51 (131)	0.013	-6.38~-0.77
Temporal	50.81 ± 13.43 (131)	54.04 ± 11.43 (131)	0.006	-5.50~-0.95
Global quadrant	47.56 ± 13.40 (524)	51.05 ± 12.60 (524)	0.001^∗^	-4.64~-2.33

*s* means standard deviation; ^∗^*P* < 0.001.

**Table 2:** Comparisons on the clinical data from A-Scan in the APAC and fellow eyes $\left(\bar{x}\pm s\right)\left(n\right)$.

	Data from A-Scan			
	APAC	Fellow	*P*	95% CI
AL ± *s*(*n*)	22.38 ± 0.82 (131)	22.38 ± 0.96 (131)	0.943	-0.09~0.09
CACD ± *s*(*n*)	2.37 ± 0.28 (131)	2.43 ± 0.25 (131)	0.051	-0.10~0.00
LT ± *s*(*n*)	4.75 ± 0.57 (131)	4.87 ± 0.47 (131)	0.021	-0.21~-0.02
LAF ± *s*(*n*)	2.13 ± 0.28 (131)	2.18 ± 0.23 (131)	0.025	-0.10~-0.01
LP ± *s*(*n*)	4.75 ± 0.29 (131)	4.86 ± 0.25 (131)	0.001^∗^	-0.16~-0.06
RLP ± *s*(*n*)	2.12 ± 0.13 (131)	2.17 ± 0.11 (131)	0.001^∗^	-0.07~-0.03

*s* means standard deviation; ^∗^*P* < 0.001.

**Table 3:** Linear regression analysis between AOD500 and other anterior segment parameters in the APAC and fellow eyes.

	AOD500					
	APAC			Fellow		
	LR equation	*R*	*P*	LR equation	*R*	*P*
IC	*Y* = 0.011 + 0.060*X*	0.137	0.001		0.056	0.160
IS		0.070	0.084		0.075	0.060
ILA		0.031	0.435	*Y* = 0.161 − 0.003*X*	0.178	0.001^∗^
ILCD	*Y* = −0.003 + 0.029*X*	0.244	0.001^∗^	*Y* = 0.049 + 0.070*X*	0.350	0.001^∗^
ICPA		0.069	0.081	*Y* = 0.089 + 0.001*X*	0.113	0.004
LCBA	*Y* = −0.007 + 0.001*X*	0.144	0.001^∗^	*Y* = 0.045 + 0.001*X*	0.158	0.045
AL		0.043	0.593	*Y* = −0.271 + 0.015*X*	0.168	0.033
CACD		0.002	0.978	*Y* = −0.108 + 0.072*X*	0.208	0.008
LT		0.023	0.774		0.087	0.273
LAF		0.029	0.719		0.013	0.870
LP		0.021	0.793	*Y* = −0.404 + 0.097*X*	0.284	0.001^∗^
RLP		0.036	0.647		0.146	0.066

^∗^ *P* < 0.001.

**Table 4:** Linear regression analysis between TIA and other anterior segment parameters in the APAC and fellow eyes.

	TIA					
	APAC			Fellow		
	LR equation	*R*	*P*	LR equation	*R*	*P*
IC	*Y* = 0.877 + 5.133*X*	0.142	0.001^∗^		0.051	0.197
IS		0.068	0.090	*Y* = 5.033 + 1.177*X*	0.078	0.050
ILA		0.035	0.380	*Y* = 13.829 − 0.265*X*	0.175	0.001^∗^
ILCD	*Y* = −0.264 + 2.419*X*	0.247	0.001^∗^	*Y* = 4.440 + 5.914*X*	0.346	0.001^∗^
ICPA		0.064	0.105	*Y* = 7.676 + 0.036*X*	0.119	0.003
LCBA	*Y* = −0.488 + 0.051*X*	0.139	0.001^∗^	*Y* = 3.545 + 0.113*X*	0.173	0.001^∗^
AL		0.048	0.547	*Y* = −19.904 + 1.143*X*	0.156	0.049
CACD		0.012	0.885	*Y* = −8.658 + 5.910*X*	0.209	0.008
LT		0.008	0.916		0.087	0.274
LAF		0.016	0.839		0.018	0.821
LP		0.002	0.977	*Y* = −32.851 + 7.992*X*	0.285	0.001^∗^
RLP		0.017	0.830	*Y* = −16.173 + 10.052*X*	0.157	0.048

^∗^ *P* < 0.001.

## Data Availability

The data used to support the findings of this study are available from the corresponding author upon request.
